# Patients’ Preferences Towards Decision Counseling for Active Surveillance After Neoadjuvant Chemoradiotherapy for Esophageal Cancer

**DOI:** 10.1245/s10434-023-14651-5

**Published:** 2023-12-15

**Authors:** Merel Hermus, Charlène J. van der Zijden, Bas P. L. Wijnhoven, Jan J. Busschbach, Sjoerd M. Lagarde, Leonieke W. Kranenburg

**Affiliations:** 1 Department of Psychiatry, Section of Medical Psychology and Psychotherapy, Erasmus University Medical Center, Rotterdam, The Netherlands; 2 Department of Surgery, Erasmus MC Cancer Institute, Erasmus University Medical Center, Rotterdam, The Netherlands

**Keywords:** Esophageal cancer, Neoadjuvant chemoradiotherapy, Active surveillance, Esophagectomy, Decision counseling

## Abstract

**Background:**

Decision counseling (DC) is offered to enable patients to reflect on their treatment preferences and to think through the consequences of alternative treatment options. However, the timing of DC is debatable. In this study, patients who underwent DC at different times were interviewed about their experiences, specifically focusing on the timing of DC.

**Methods:**

Patients with locally advanced esophageal cancer eligible for participation in a prospective cohort study on active surveillance (SANO-2 study) were offered DC either before or after neoadjuvant chemoradiotherapy (nCRT). Structured interviews were conducted by phone 1 week after DC, and responses were analyzed using frequency counts for the answers to set response categories. The primary outcome was the preferred time to receive DC, while the secondary outcome was the overall experience of patients with DC.

**Results:**

Overall, 40 patients were offered DC between 2021 and 2023. Patients who had counseling before the start of nCRT (*n* = 20) were satisfied with the timing of DC. Of the 20 patients who had DC after nCRT, 6 would have preferred counseling at an earlier time point. Patients who had DC both before or after the completion of nCRT reflected positively on DC.

**Conclusion:**

It is recommended to introduce the option of DC as early as possible and discuss with the patient at which moment during the decision-making process they prefer to discuss all treatment options more extensively.

Active surveillance is being investigated as an alternative to standard esophagectomy in patients with esophageal cancer. The *Surgery As Needed for Oesophageal* cancer (SANO) trial aims to evaluate the non-inferiority of active surveillance in patients with a clinically complete response (cCR) after neoadjuvant chemoradiotherapy (nCRT).^1,2^ The decision between esophagectomy or active surveillance can be challenging for both doctors and patients as there is uncertainty about the efficacy and safety of active surveillance. Until the results of the SANO trial are available, the treatment decision strongly depends on the preferences and values of the individual patient.

Pending evidence that supports the wider introduction of active surveillance, it is important to gain insight into the experiences and needs of patients about the information provision of this experimental treatment. Therefore, decision counseling (DC) was integrated in the SANO-2 prospective cohort study, an extension study of the SANO trial.^3^ Within this study, DC was offered by a psychologist or medical researcher to all patients who were for nCRT followed by esophagectomy. This method allows clinicians and patients to identify patients’ personal values and preferences, aiding in the selection of the most appropriate treatment option.^4^ The timing of DC is debatable as the decision-making process can span several months, starting from initial diagnosis until active surveillance becomes a viable option following cCR after nCRT. One can argue that DC might be offered before initial treatment, as this ensures that patients have the essential information to make an informed decision about their treatment early on.^5,6^ On the other hand, DC may also be offered at a later time since it prevents patients from being overwhelmed with an excessive amount of information. In this scenario, clinicians act as information filters, providing only the essential and relevant information at each stage of the decision-making process.^7^ What adds to the complexity is that for most patients, active surveillance will not be an option as only up to one-third of patients are expected to achieve a cCR after nCRT (unpublished data).

The aim of this study was to investigate the preferred timing of DC and to explore patients’ experiences with DC when they have to face the decision between standard esophagectomy and active surveillance. These findings provide insight into how DC is experienced within this context and can contribute to the development of a decisional aid tailored to the needs of patients with esophageal cancer.

## Methods

### Study Design

This study was performed in the context of the SANO-2 study, a multicenter prospective observational cohort study that monitors the safety and effectiveness of active surveillance while awaiting the results of the randomized trial.^3^ The study was initiated by the Erasmus MC Cancer Institute and was conducted at 11 hospitals in The Netherlands. The study was approved by the Medical Ethical Committee of the Erasmus MC (MEC 2021-0068) and was registered at ClinicalTrials.gov (NCT04886635).^8^ All patients provided written informed consent.

### Patients

Operable patients above 18 years of age who underwent or were planned to undergo nCRT according to the CROSS regimen followed by surgical resection for histologically proven adenocarcinoma or squamous cell carcinoma of the esophagus or junction at the Erasmus MC Cancer Institute were offered DC.^3^

### Procedures: Decision Counseling (DC) and Evaluation

Patients who visited an upper gastrointestinal surgeon at the start of their treatment were offered the possibility of DC with a psychologist or medical researcher (MH or CZ). Both researchers were trained to elicit, explore, and discuss patients’ preferences in such a way that patients were enabled to think through the consequences of each treatment option for them personally. If patients expressed no need for counseling prior to the start of nCRT, the option of DC after completion of nCRT was offered. As a result, two groups were created: patients who had DC before or after nCRT.

During DC, the pros and cons of both surgery and active surveillance were discussed. Furthermore, the pros and cons were considered in relation to the patients’ preferences. For example, questions such as ‘what is important to you in life and how would active surveillance/surgery impact that?’ would help the patient oversee the consequences of the treatment decision. At the end of the counseling, patients were asked if they already had a treatment preference, and if so, what the reason was for this preference. This allowed the researcher to verify the patient’s understanding of treatment consequences. If the patient did not have a preference yet, the researcher asked what was needed to help them make a decision. Patients were informed that they would be contacted 1 week after DC took place, to evaluate the counseling and to verify whether the patient had made a treatment decision. Structured interviews were used for evaluation of DC. All questions had set response categories and patients were encouraged to elaborate on their answers. The interviews to evaluate DC were conducted by telephone by the opposed researcher (MH or CZ) who offered DC 1 week before, in order to ensure objectivity.

### Study Outcomes and Statistical Analyses

The primary outcome was the preferred time point to offer DC, while the secondary outcome was the overall experience of patients with DC, including patients’ reflection on DC and its usefulness in aiding decision making. Responses were analyzed using frequency counts for the answers to the set response categories and by grouping answers to the open questions into themes. Baseline characteristics were presented with frequency (percentage) for categorical variables or median (minimum–maximum) for continuous variables.

## Results

**Fig. 1:**
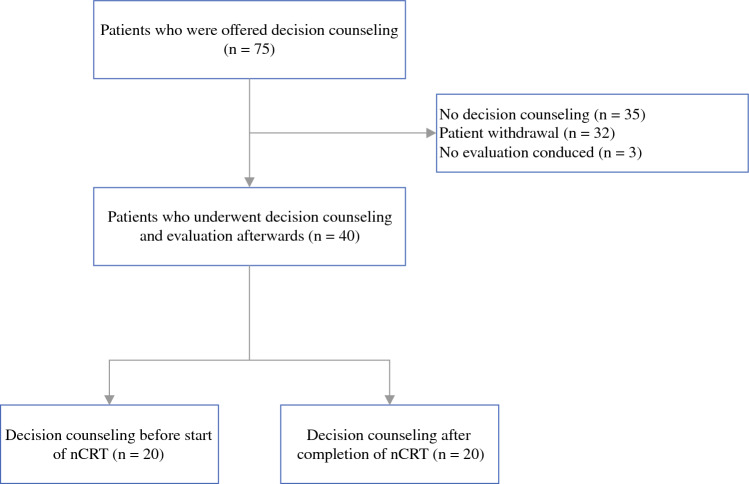
Study flowchart of patients who were offered decision counseling. *nCRT* neoadjuvant chemoradiotherapy

Between June 2021 and February 2023, 75 patients were offered DC before the start or after completion of nCRT; 32 of the 75 patients (42.7%) indicated that they had no need for counseling because they had already made a treatment decision (*n* = 30) or they had sufficient information to make a treatment decision at a later time (*n* = 2). All other patients agreed to participate, but three patients were not interviewed because they could not be reached by telephone after three attempts. This resulted in 40 patients being available for evaluation of the primary and secondary outcomes (Fig. 1).

Median age of all patients was 68 years (interquartile range 64–73) and most patients had adenocarcinoma (85%) and were classified as WHO performance status 0–1 (85%) (Table 1). The results of the structured interviews are presented in Table 2 and are discussed below.

**Table 1:** Baseline characteristics of patients who were offered decision counseling before the start or after completion of neoadjuvant chemoradiotherapy

Characteristics	All patients [*N* = 40]	DC before nCRT	DC after nCRT [*n* = 20]
		[*n* = 20]	
Sex			
Male	22 (55)	13 (65)	9 (45)
Age, years [median (IQR)]	68 (64–73)	69.5 (66.3–73)	67 (64–75)
Tumor type			
Adenocarcinoma	34 (85)	19 (95)	15 (75)
Squamous cell carcinoma	6 (15)	1 (5)	5 (25)
Clinical T category			
cT2	16 (40)	9 (45)	7 (35)
cT3	20 (50)	10 (50)	10 (50)
cTx	4 (10)	1 (5)	3 (15)
Clinical N category			
cN0	17 (42.5)	9 (45)	8 (40)
cN1	17 (42.5)	9 (45)	8 (40)
cN2	5 (12.5)	2 (10)	3 (15)
cNx	1 (2.5)	–	1 (5)
WHO performance status			
0–1	34 (85)	18 (90)	16 (80)
2	1 (2.5)	–	1 (5)
Unknown	5 (12.5)	2 (10)	3 (15)

Data are expressed as *n *(%) unless otherwise specified

*DC* decision counseling, *nCRT* neoadjuvant chemoradiotherapy, *IQR* interquartile range

**Table 2:** Overview of the questionnaire for evaluation of decision counseling collected 1 week after decision counseling was performed

Answer options	Before the start of nCRT							After the completion of nCRT						
	Q1	Q2	Q3	Q4	Q5	Q6	Q7	Q1	Q2	Q3	Q4	Q5	Q6	Q7
Very positive/yes totally	4	8	5	5	2	1		5	10	5	4			
Positive/yes	16	11	14	11	3	1		13	10	14	9	3	2	6
Somewhat				1	2						1	1		
Neutral		1	1	1		3	2	2		1			5	1
Negative/no				1	8	11	15				2	10	12	12
Very negative/not at all				1	5	3	2					6	1	1
Not applicable/undecided						1	1				4			

*nCRT* neoadjuvant chemoradiotherapy,* Q* question

Q1: How do you reflect on the decision counseling conversation?

Q2: Was the information clear?

Q3: Was sufficient attention paid to your own questions and considerations during decision counseling?

Q4: Were you able to make a treatment decision after decision counseling?

Q5: Did you experience doubts about the treatment decision?

Q6: Would you rather have had decision counseling with your own treating doctor (i.e., surgeon)?

Q7: Would you rather have had decision counseling at another time point?

Q1–Q3 refer to evaluation of decision counseling

Q4–Q5 refer to outcomes following decision counseling

Q6–Q7 refer to patient preferences regarding the timing and person who provides decision counseling

### Patient Preferences Regarding the Timing and Person Who Offered DC

Only 2 of 20 patients who had DC before nCRT would have preferred DC from their surgeon instead of the independent psychologist or medical researcher (“*My surgeon knows me better*”). The same was found in 2 of 20 patients who had DC after completion of nCRT (“*Talking to your own surgeon gives me more confidence”)*. Most patients were indifferent whether DC should be provided by an independent person or their treating surgeon. Three patients explicitly preferred counseling from an independent person (“*It was pleasant to feel less rushed, the doctor is always so busy”*).

All patients who had DC before the start of nCRT confirmed in hindsight that this had been the right moment for them. Six of 20 patients who had DC after completion of nCRT expressed in hindsight that they would have preferred DC at another time point. Among these six patients, three patients would have preferred DC before the start of nCRT to better understand what to expect, while two would have preferred DC at a later time point when recovered from nCRT. One patient was uncertain about the optimal timing for DC due to an overload of information (*“The timing now was not ideal because there was an overload of information. I don’t know when it would be a better timing though”*).

### Outcomes Following DC

Of the 20 patients who had DC before the start of nCRT, 16 opted for active surveillance (i.e., participation in the SANO-2 study). One patient decided to undergo esophagectomy, while three patients remained undecided at the moment of evaluation, having the intention to make the treatment decision after completion of nCRT (*“I first want to wait on the results of the PET/CT scan after nCRT, only then I can decide if I want to participate in the SANO-2 study or not”*)*.*

Of the 20 patients who had DC after completion of nCRT, 19 opted for active surveillance and one patient remained undecided at the moment of evaluation.

### Evaluation of DC

All patients reflected positively on DC. They thought the information was clear and that their questions were adequately addressed. Four patients who had DC after completion of nCRT indicated that although their preference before DC was similar as after DC, they reflected positively on DC (*“I already received all information from the doctor and made my decision, but I appreciated that there was extra time to discuss the pros and cons of both treatment options”*).

## Discussion

This study explores the timing of DC for patients with esophageal cancer who were given the opportunity to choose active surveillance after completion of nCRT, as an alternative to standard esophagectomy. Patients who had DC before nCRT were satisfied with the moment that DC was offered, whereas some patients who had DC after completion of nCRT would have preferred counseling earlier or even a later time point. Overall, patients were positive about the option of DC because it facilitated making a more balanced treatment decision.

Although it is without question that patients should be enabled to arrive at a well-informed decision on future treatment, clinical practice may be somewhat different. Shared decision making requires time and specific communicational skills from a doctor to guide patients in making a well-informed decision. This is particularly true when a standard invasive treatment (i.e., surgery) is discussed and offered against a non-surgical or delayed surgical strategy (i.e., active surveillance).^1–3,9^ In the present setting of active surveillance for esophageal cancer, data from randomized studies on long-term outcomes are still lacking, and consequently cannot be completely taken into consideration. Second, personal opinions or intuitive predictions of the doctor on the long-term outcomes, which have yet to be confirmed in the randomized controlled trials, can lead to bias. To counteract this bias, DC was offered by an independent psychologist or medical researcher, who were trained to mitigate this bias.

When non-inferiority of active surveillance can be demonstrated in the future, DC will change in two ways. First, the trade-off discussion is no longer the subjective risk of active surveillance versus deterioration of quality of life associated with surgery, because trial results will provide information on risk ratios. Second, DC will no longer be offered within the context of a study setting but will have to be integrated into the standard-of-care process. This means that DC will no longer be offered by study staff (i.e., medical researcher or psychologist) but will be handled over to the patients’ healthcare providers. It is likely that the physician will be the one providing such counseling in the future, as he/she discusses all possible treatment options with the patient. However, in some cases, referral to a counselor or psychologist may be needed; for instance, when a patient gets stuck in the decision-making process, does not seem to fully comprehend the consequences of a particular treatment choice, or feels like their physician is ‘too busy’ to elaborate on treatment options.

To help physicians facilitate the process of shared decision making and support patients in making informed decisions, a decision aid can be a helpful tool. Decision aids exist in multiple forms (online, print, video) and aim to inform patients about available options, encourage them to active engagement with the decision-making process, and to help patients think through what is important to them in order to make choices that reflect their values and preferences.^10^ Furthermore, even if active surveillance is not established as non-inferior, some patients may still opt for this treatment option. Consequently, surgeons may benefit from additional resources to effectively manage the impact of patients’ personal preferences on their decision-making process. A decision aid enables the physicians to tailor the counseling approach for maximum efficiency and make the most of the available time. Thus far, decision aids with active surveillance as a treatment option have been developed mostly for prostate cancer, and interventions are needed for esophageal cancer.^11^ For example, practical and personal considerations play a role in the treatment decision, as mentioned by some patients:

(“Also practical matters played a role, like arranging taxi rides to the hospital for each response evaluation if I chose active surveillance”).

(“I am 76 years old, and I think that recovery from surgery takes longer at an older age. Therefore, I prefer to postpone surgery if that is possible”).

(“I am used to visiting the hospital a lot, so the possible disadvantage of active surveillance (regular visits to the hospital), is no problem for me”).

The present study suggests that patients do not experience it as burdensome if all information is provided before the start of therapy (in this case, nCRT). However, for most patients, active surveillance will not be an option as only up to one-third of patients are expected to achieve a cCR after nCRT. It is questionable how burdensome it is for these patients to find out that active surveillance is no longer an option and that esophagectomy is indicated to pursue curation. We have to keep in mind that these thoughts specifically apply to patients with esophageal cancer and that other cancer types perhaps do not allow to use a decision aid at a later time, because the treatment decision has to be made shortly after diagnosis. Therefore, the treatment of locally advanced esophageal cancer (if active surveillance will be added as an alternative treatment option) creates a unique setting where a decision aid can be deployed when the patient needs it. In addition, by initiating DC prior to the start of nCRT, patients have the opportunity to express their preference to postpone the counseling to a moment after the completion of neoadjuvant therapy.

An important strength of this study is that all patients were offered DC with a trained psychologist or medical researcher and that the structured interviews 1 week after DC were conducted by the opposed researcher, to ensure objectivity. In addition, a structured interview was used in order to gather information and experiences on equal fronts for patients who had DC before the start or after completion of nCRT, respectively. Some limitations should also be considered. DC was offered to all patients with esophageal cancer who qualified for participation in the SANO-2 study, and some had already signed the informed consent form before the start or after completion of nCRT, which resulted in a certain selection bias. Moreover, this study describes the results of one single center, which may limit the generalizability. The sample size was limited as only 40 patients were included. Nevertheless, the findings provide a good overview of patients’ preferences regarding the optimal timing for providing all information needed to make a treatment decision.

## Conclusion

This study provides insight into patients’ preferred timing of DC when a potentially new treatment strategy (i.e., active surveillance) can be chosen. This insight will contribute to the development of a decision aid tailored to the needs of patients with esophageal cancer.
